# Crystal structure and functional characterization of a light-driven chloride pump having an NTQ motif

**DOI:** 10.1038/ncomms12677

**Published:** 2016-08-24

**Authors:** Kuglae Kim, Soon-Kyeong Kwon, Sung-Hoon Jun, Jeong Seok Cha, Hoyoung Kim, Weontae Lee, Jihyun F. Kim, Hyun-Soo Cho

**Affiliations:** 1 Department of Systems Biology and Division of Life Sciences, Yonsei University, 50 Yonsei-ro, Seoul 03722, Republic of Korea; 2 Department of Biochemistry and Division of Life Sciences, Yonsei University, 50 Yonsei-ro, Seoul 03722, Republic of Korea; 3 Strategic Initiative for Microbiomes in Agriculture and Food, Yonsei University, 50 Yonsei-ro, Seoul 03722, Republic of Korea

## Abstract

A novel light-driven chloride-pumping rhodopsin (ClR) containing an ‘NTQ motif' in its putative ion conduction pathway has been discovered and functionally characterized in a genomic analysis study of a marine bacterium. Here we report the crystal structure of ClR from the flavobacterium *Nonlabens marinus* S1-08^T^ determined under two conditions at 2.0 and 1.56 Å resolutions. The structures reveal two chloride-binding sites, one around the protonated Schiff base and the other on a cytoplasmic loop. We identify a ‘3 omega motif' formed by three non-consecutive aromatic amino acids that is correlated with the B–C loop orientation. Detailed ClR structural analyses with functional studies in *E. coli* reveal the chloride ion transduction pathway. Our results help understand the molecular mechanism and physiological role of ClR and provide a structural basis for optogenetic applications.

## Abstract (web summary)

The atypical rhodopsin ClR from flavobacterium *Nonlabens marinus* is a light-driven chloride-pumping protein. Here, the authors show that ClR crystal structure presents two chloride ion-binding sites, proposing a molecular pathway for ion transport by this light-driven pump.

## Graphical abstract

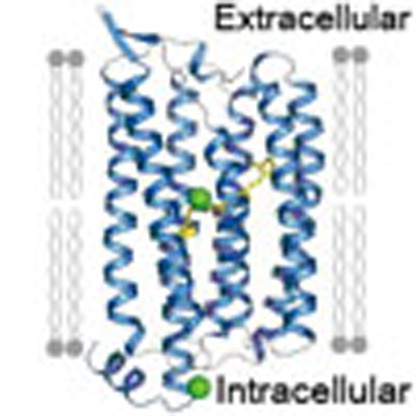

Microbial light-driven ion-pumping rhodopsins generate the membrane potential in response to light by actively transporting ions across the cell membrane^1^. In the light-adapted state, the chromophore of the ion pump is all-*trans* retinal bound to a lysine residue via the Schiff base linkage^2^. Absorption of light induces the isomerization of all-*trans* retinal to the 13-*cis* conformation, which leads to structural changes in the protein that result in ion transport. Archetypal microbial light-driven, ion-pumping rhodopsins include bacteriorhodopsin (BR) and halorhodopsin (HR), which are found in halophilic archaeal species living in extremely high-salt environments^3^,^4^. BR is an outward proton pump rhodopsin and HR is an inward chloride pump rhodopsin. During the BR photocycle, the proton on the Schiff base is transferred to the proton acceptor Asp85 (D85), and then the proton donor Asp96 (D96) re-protonates the Schiff base for the next photocycle^5^,^6^. These two residues of BR along with Thr89 (T89) form a highly conserved DTD motif in the third transmembrane helix. In HR, the proton acceptor and donor residues of BR, Asp85 and Asp96 are changed to the neutral residues, forming a TSA (Thr, Ser, and Ala) motif. The crystal structure of HR showed that a chloride ion binds at the protonated Schiff base (PSB)^7^.

Metagenomic studies of marine microorganisms have demonstrated that ion-pumping rhodopsins are also broadly present in the domain of bacteria. In 2000, a BR-like proton-pumping rhodopsin from a proteobacterium was identified and named proteorhodopsin^8^. This showed that light-driven energy accumulation via ion-pumping rhodopsins is widely used among marine bacteria. Furthermore, in 2013, a novel class of microbial rhodopsins was found in *Nonlabens* (*Donghaeana*) *dokdonensis* DSW-6^T^ and other marine flavobacteria^9^. These rhodopsins were dubbed NQ rhodopsins because they have Asn (N) and Gln (Q) at the positions of the proton acceptor and donor residues of BR^9^. Phylogenetic analysis indicated that NQ rhodopsins are evolutionarily distinct from other microbial rhodopsins^9^ and that NQ rhodopsins can be further classified as NDQ or NTQ rhodopsins depending on the residue at the position of Thr89 of BR^10^. An NDQ rhodopsin was shown to function as a light-driven outward sodium pump (thus called NaR)^11^, and the crystal structures of NaR from *Dokdonia eikasta*^12^ (previously reported as *Krokinobacter eikastus*) NBRC 100814^T^ were reported recently^13^,^14^. In contrast, the NTQ rhodopsin functions as a light-driven inward chloride pump (called ClR)^10^. In ClRs, none of the residues involved in chloride ion binding in HR is conserved^15^, and a spectroscopic study of ClR suggested both conserved and unique mechanisms of chloride transport compared with that of HR^16^. Although studies performed in halophilic archaea made appreciable progress in understanding the molecular nature of the microbial light-driven chloride pump, the molecular mechanisms of chloride transport are largely unknown.

Here we report the atomic crystal structure of ClR from the flavobacterium *Nonlabens marinus* S1-08^T^. The structure reveals two chloride ion-binding sites, one at the active centre of the pump and the other on a loop in the cytoplasmic side, suggesting the pathway of chloride ion movement in ClR. The structure also shows that the chloride ion at PSB directly interacts with the conserved residues Asn98 and Thr102 of the NTQ motif as well as PSB. The internal ClR structure and biochemical assays identify amino-acid residues important for chloride ion transport. Therefore, our results demonstrate the unique structural features of ClR and provide a basis to understand the mechanism of chloride ion transport through this new light-driven chloride pump in marine bacteria.

## Results

### Structure determination of ClR

We crystallized ClR from *N. marinus* under two different sets of conditions using the lipid cubic phase method^17^. Type A crystals, obtained at pH 6.0, diffracted to 1.56 Å and type B crystals, obtained at pH 4.5, diffracted to 2.0 Å (Table 1). In both types of crystals, there is one molecule of ClR in the asymmetric unit. The structure was determined by molecular replacement using the NaR structure (PDB ID: 3X3B)^13^ as a search model. The type A and type B crystal structures were refined to a *R*_work_/*R*_free_ of 15.40/19.38% and 17.84/22.24%, respectively. The final type A and type B crystal structures contained ClR residues 2–265 and 1–266, respectively. The electron density maps of type A and B crystals showed clear densities for the side chains of most residues and type A and B crystals showed different crystal-packing pattern (Supplementary Fig. 1). Because ClR showed similar light-induced pumping activity with Cl^−^ and Br^−^ (ref. 10), it was possible to locate halide ion-binding sites in the ClR structure using an anomalous signal. We prepared both type A and B crystals supplemented with NaBr under the conditions used to obtain the native crystals (Methods) and collected diffraction data at an absorption peak wavelength of Br^−^, 0.9197 Å (Table 1).

### Overall structure of ClR and chloride ion-binding sites

**Figure 1:**
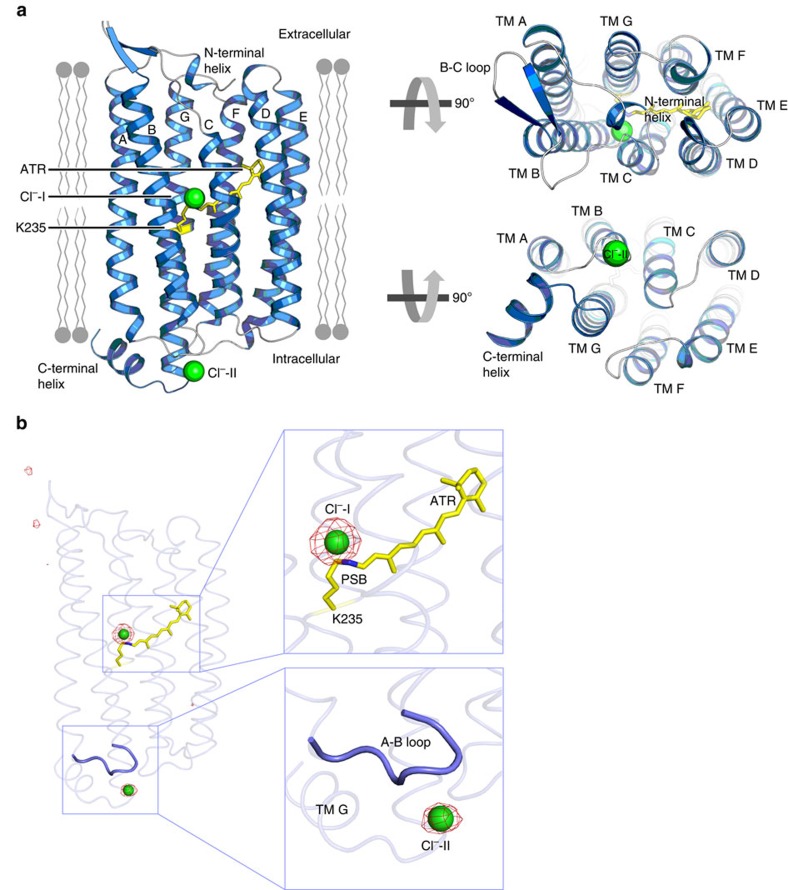
Crystal structure of the ClR. (**a**) ClR structure in a ribbon model. Transmembrane helices A–G (TM A–TM G) are indicated and the putative membrane parts are drawn schematically. (**b**) Anomalous difference Fourier map of Br^−^ shown as red meshes (contoured at 5.0*σ*). Magnified views of the peaks of the map are shown in boxes. Two chloride ions bound to ClR structure are depicted by green spheres. All-*trans* retinal (ATR), linked covalently to K235, is shown in a yellow stick model.

ClR shares higher sequence identity with NaR from *D. eikasta* (35%) than those with HRs from *Halobacterium salinarum* (HsHR, 19%) and *Natronomonas pharaonis* (NpHR, 17%)^18^ (Supplementary Fig. 2). A phylogenetic analysis showed that ClRs form a distinct clade with NaRs containing the NDQ motif^10^. The architecture of ClR is also closer to that of NaR than that of HR (Fig. 1a and Supplementary Fig. 2), confirming the notion that ClR and HR, the bacterial and halophilic archaeal light-driven chloride pumps, have evolved independently^10^. The ClR structure comprises a short N-terminal helix, seven transmembrane helices (TM A–TM G), a C-terminal helix and three loops connecting the transmembrane helices on both extracellular and cytosolic sides (Fig. 1a and Supplementary Fig. 2). The N-terminal helix was also revealed in the crystal structures of *D. eikasta* NaR^13^,^14^.

Very strong peaks in the anomalous difference Fourier map of Br^−^ showed two halide ion-binding sites in the ClR structure that are coordinated well by the ClR protein; therefore, we modelled chloride ions into these sites (Fig. 1b). The first chloride ion (Cl^−^-I) is located in the vicinity of the Schiff base at the active centre of ClR and the second chloride ion (Cl^−^-II) is positioned on the loop connecting TM A and TM B (A–B loop) on the cytoplasmic side (Fig. 1a,b).

**Figure 2:**
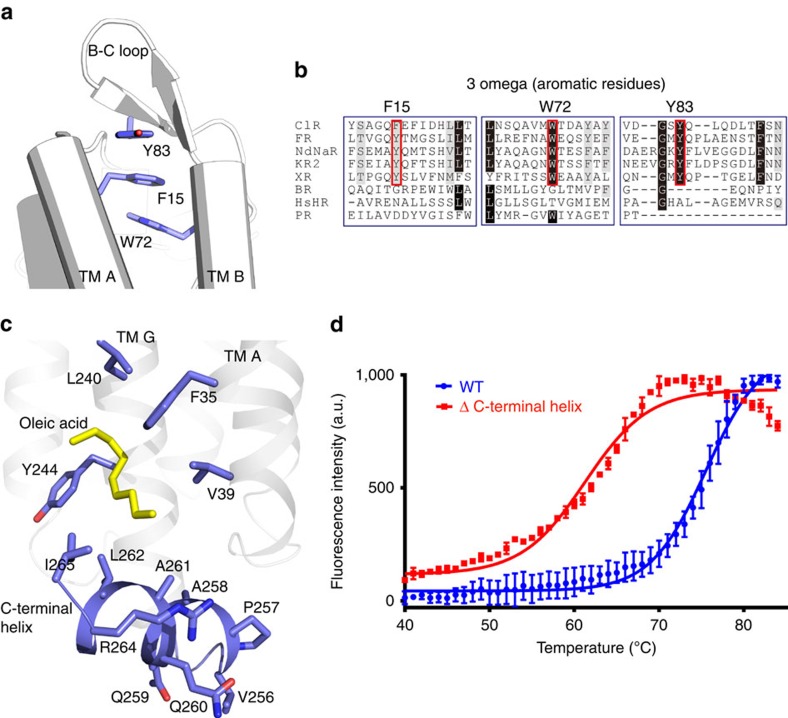
Structural features of ClR. (**a**) 3 omega motif of ClR formed by *π*-stacking interactions of the side chains of aromatic residues in TM A (F15), TM B (W72) and B–C loop (Y83). (**b**) Sequence alignment around the 3 omega motif. Conserved aromatic residues forming the 3 omega motif are indicated with red open rectangles. ClRs from *N. marinus* and *Fulvimarina pelagi* (FR), sodium-pumping rhodopsins from *Nonlabens dokdonensis* (NdNaR) and *Dokdonia eikasta* (*Krokinobacter eikastus*) (KR2), xanthorhodopsin (XR), BR, HR and proteorhodopsin (PR). Identical and similar residues are highlighted in black and grey, respectively. (**c**) Interaction between the C-terminal helix (blue) and oleic acid (yellow). Hydrophobic residues in TMs A and G, which interact with the oleic acid are also depicted in blue. (**d**) Fluorescence thermal stability profiles of wild-type (WT) and ΔC-terminal helix ClRs. Tm values are 75 °C for WT and 61 °C for ΔC-terminal helix ClR. The curves are representative of three experiments and the error bars represent the mean±s.d. a.u., arbitrary units.

The B–C loop of ClR, which connects TM B and TM C, adopts a different conformation than that adopted by the B–C loop of HR. The orientation of the B–C loop of ClR is towards TMs A and B, whereas HR B–C loop is oriented towards TMs D and E (Fig. 1a and Supplementary Fig. 3a). The different orientations of the B–C loop between NaR and BR were also pointed out in the previous report of the NaR structure^13^. We find that the side chains of three nonconsecutive aromatic amino acids in ClR, Phe15 (in TM A), Trp72 (in TM B) and Tyr83 (in B-C loop), stack with one another, tethering the B–C loop in the direction of TMs A and B (Fig. 2a). These aromatic amino acids are highly conserved in NTQ-, NDQ- and xanthorhodopsin-type light-driven ion pumps, which are evolutionarily closer to one another than to other microbial rhodopsins, but absent in other rhodopsin pumps (Fig. 2b and Supplementary Fig. 3b,c). This suggests that the orientation of the B–C loop in microbial light-driven ion pumps may be correlated with the presence of these aromatic amino-acid residues. We refer to the structural motif formed by these conserved residues as the ‘3 omega motif', representing three aromatic amino-acid residues. A fluorescent thermal stability assay of F15A, W72A and Y83A mutants using the thiol-specific fluorochrome *N*-[4-(7-diethylamino-4-methyl-3-coumarinyl)phenyl]maleimide (CPM) showed that the mutants were slightly unstable compared with the wild-type protein (Supplementary Fig. 3d), suggesting a structural role of the 3 omega motif.

The structure reveals that ClR has an additional α-helix, the ‘C-terminal helix', after TM G (Figs 1a and 2c). In other microbial rhodopsin pumps of known structures, the counterparts of the C-terminal helix of ClR cannot be observed or are only partially observed; probably because they are disordered in the crystals. The C-terminal helix of ClR is reminiscent of amphipathic helix 8 of the eukaryotic class A G-protein-coupled receptors, which connects the membrane part of the G-protein-coupled receptors to the cytosolic domains^19^. The C-terminal helix of ClR is also amphipathic and its hydrophobic interface, formed by Pro257, Ala258, Ala261, Leu262 and Ile265, interacts with an oleic acid located on TM A (Fig. 2c). To investigate the role of the C-terminal helix in ClR, we prepared the C-terminal helix-deletion mutant Δ255–272. This mutant could be expressed and purified like the wild-type ClR, but the CPM assay showed that C-terminal helix-deleted mutant's Tm (melting temperature) value is much lower than that of the wild-type ClR (wild type, 75 °C; ΔC-terminal helix, 61 °C; Fig. 2d) suggesting that the C-terminal helix might be important for the stability of ClR, presumably through interactions with the hydrophobic region of the membrane.

### Structure at the Schiff base environment

**Figure 3:**
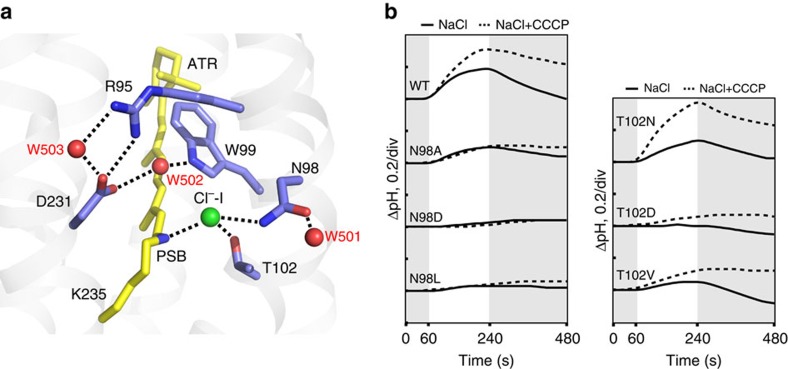
ClR structure at the PSB region. (**a**) Detailed structure of ClR at PSB. Chloride ion and water molecules are shown by green and red spheres, respectively, and hydrogen bonds are depicted in dashed lines. (**b**) Light-induced pump activities of wild-type (WT) ClR and its mutants at Asn98 and Thr102. The pumping activities are measured in NaCl (lines) and NaCl with carbonyl cyanide *m*-chlorophenylhydrazone (CCCP; dashed lines). Grey and white sections indicate dark and light conditions, respectively.

The retinal adopts the all-*trans* conformation in the ClR structure. A detailed analysis of the ClR structure near PSB reveals interactions among Cl^−^-I, PSB and the conserved residues of the NTQ motif (Fig. 3a). Cl^−^-I, which is located 3.1 Å away from PSB, interacts directly with PSB by electrostatic and hydrogen-bonding interactions. The conserved Asn98 and Thr102 of the NTQ motif are hydrogen bonded with Cl^−^-I, stabilizing the coordination of Cl^−^-I. Three water molecules (W501, W502 and W503) that interact with amino-acid residues are well resolved around Cl^−^-I and PSB. W501 interacts with OD-1 of Asn98, the other side of the side chain, stabilizing the interaction of ND-2 of Asn98 with Cl^−^-I. At the more extracellular side, W502 and W503 simultaneously interact with the side chains of Asp231 and Arg95, the charged amino-acid pair conserved in all ion-pumping microbial rhodopsins. W502, located between Cl^−^-I–PSB and Asp231-Arg95, is 3.8 Å away from Cl^−^-I and 4.2 Å away from PSB. Thus, Asp231–Arg95 is not connected to Cl^−^-I–PSB by a water-mediated hydrogen-bonding network. The detailed structure of ClR around PSB shows that although the Cl^−^-I-binding site in ClR is similar to the previously identified chloride-binding site of HR, the following aspects are different: (1) in ClR, Cl^−^-I forms a direct hydrogen bond with PSB; (2) in HR, three well-resolved water molecules form a hydrogen bond network with Asp238–Arg108 (in HR from *H. salinarum*) and chloride ion-PSB, whereas in ClR, there is no hydrogen bond network of water molecules connecting Asp231–Arg95 to Cl^−^-I–PSB (Supplementary Fig. 4). A spectroscopic study of the NTQ rhodopsin from *Fulvimarina*, named FR, suggested that chloride binding to FR is much weaker than that to HR^16^. The ClR structure suggests that the absence of a water-mediated hydrogen-bonding network between the conserved Asp–Arg and Cl^−^-I–PSB might be a possible reason why chloride binding to the NTQ rhodopsin is weaker than that to HR.

The retinal conformation of ClR around PSB is different from that of HR (Supplementary Fig. 4a,b). Comparison of the retinal structure of ClR with those of other microbial rhodopsins suggests the retinal configuration of ClR is more similar to those of NaR, XR and the channel rhodopsin than those of BR, HR and proteorhodopsin (Supplementary Fig. 4c,d). A recent crystallographic study combined with a spectroscopic analysis indicated that a low dose of X-ray during data collection may modify the conformation of the active site of BR^20^. The radiation dose absorbed by the ClR crystals during data collection was modest (0.04–0.12 MGy). However, we do not rule out the possibility that the retinal chromophores in microbial rhodopsins are partially modified during the data collection.

Because Asn98 and Thr102 are the only residues of ClR directly interacting with Cl^−^-I, we investigated whether the interaction between Asn98 or Thr102 and Cl^−^-I is essential for the chloride-pumping activity of ClR. We expressed several ClR mutants (N98A, N98D and N98L for Asn98; and T102D, T102N and T102V for Thr102) in *Escherichia coli* cells. The expression levels of the mutant proteins in *E. coli* were similar to that of the wild-type protein. We monitored light-induced pH changes in *E. coli* cell suspensions to assess the chloride-pumping activities. In most mutants, except T102N, the chloride-pumping activities were eliminated or substantially decreased (<50% of the wild-type ClR; Fig. 3b), indicating that Asn98 and Thr102 play significant roles in chloride pumping. Of the mutants, T102N retained ∼70% of its pumping activity compared with the wild-type protein, whereas T102D completely lost its pumping activity. T102N's absorption spectra showed a blue shift in 1.5 M NaCl (*λ*_max_ from 562 to 529 nm) suggesting that a chloride ion can bind at PSB but T102D's absorption spectra did not change in 1.5 M NaCl (Supplementary Fig. 5a). We crystallized T102N and T102D proteins and collected diffraction data (Table 1). The T102N crystal structure shows that ND-1 of the mutated Asn is hydrogen-bonded to Cl^−^-I, as is OG-1 of Thr102 in the wild type (Supplementary Fig. 5b). In the T102D crystal structure, on the contrary, the side chain of the introduced Asp interacts with both PSB and Asn98, preventing the binding of a chloride at the Cl^−^-I site. Taken together, these results show that the direct hydrogen bond between Asn98/Thr102 (Asn98 and Thr102) and Cl^−^-I is crucial for the binding of Cl^−^-I, as well as for the chloride-pumping activity of ClR. We thus speculate that the decreased ion translocation might have arisen from the weakened binding affinity between the mutant ClRs and chloride.

### Second chloride ion-binding site

**Figure 4:**
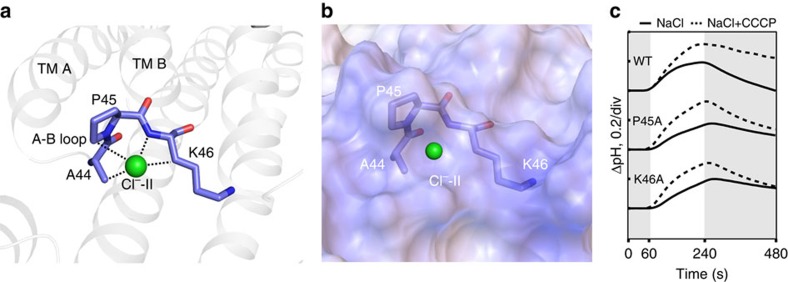
Chloride ion-binding site on the cytoplasmic loop of ClR. (**a**) Coordination of Cl^−^-II (green sphere) by Ala44, Pro45 and Lys46. Dotted lines represent hydrogen-mediated bonds. (**b**) Surface electrostatic potential around Cl^−^-II-binding site. (**c**) Light-induced pump activities of wild-type (WT) ClR and its mutants at Pro45 and Lys46. The pumping activities are measured in NaCl (lines) and NaCl with carbonyl cyanide *m*-chlorophenylhydrazone (CCCP; dashed lines). Grey and white sections indicate dark and light conditions, respectively.

The anomalous difference Fourier maps of Br^−^ from both type A and B crystals supplemented with NaBr had a strong and clear peak of electron density for Cl^−^-II (Fig. 1b), showing a second chloride ion-binding site on the cytosolic surface of ClR. At the Cl^−^-II-binding site, the chloride ion is coordinated by Ala44, Pro45 and Lys46 on the A–B loop (Fig. 4a). Cl^−^-II is hydrogen-bonded with the backbone amide nitrogen of Lys46. The aliphatic hydrogens from CB of Ala44, CD of Pro45 and CB of Lys46 partly enclose Cl^−^-II and appear to stabilize its binding. The electrostatic potential around the Cl^−^II-binding site shows a strong positive patch, which may also stabilize chloride binding (Fig. 4b). The A–B loop in HR from *H. salinarum* contains three arginine residues (Arg52, Arg55 and Arg58; Supplementary Fig. 2) that form a highly positively charged surface patch; however, chloride binding was not observed^7^. On the contrary, in the crystal structure of HR from *N. pharaonis*, the second chloride-binding site was identified on the cytosolic side near helix B (ref. 18). To observe whether the binding of a chloride to the Cl^−^II-binding site is necessary for the light-driven chloride-pumping activity of ClR, we prepared the mutants P45A and K46A. The pumping activities of these *E. coli*-expressed mutant proteins were similar to the activity of the wild-type ClR (Fig. 4c), suggesting that the binding of a chloride to the Cl^−^II-binding site might not be required for the pumping activity of ClR. Together, the binding site of a chloride ion on the cytosolic side of ClR suggests a potential chloride transfer pathway to the cytoplasmic side.

### Chloride transduction pathway in ClR

**Figure 5:**
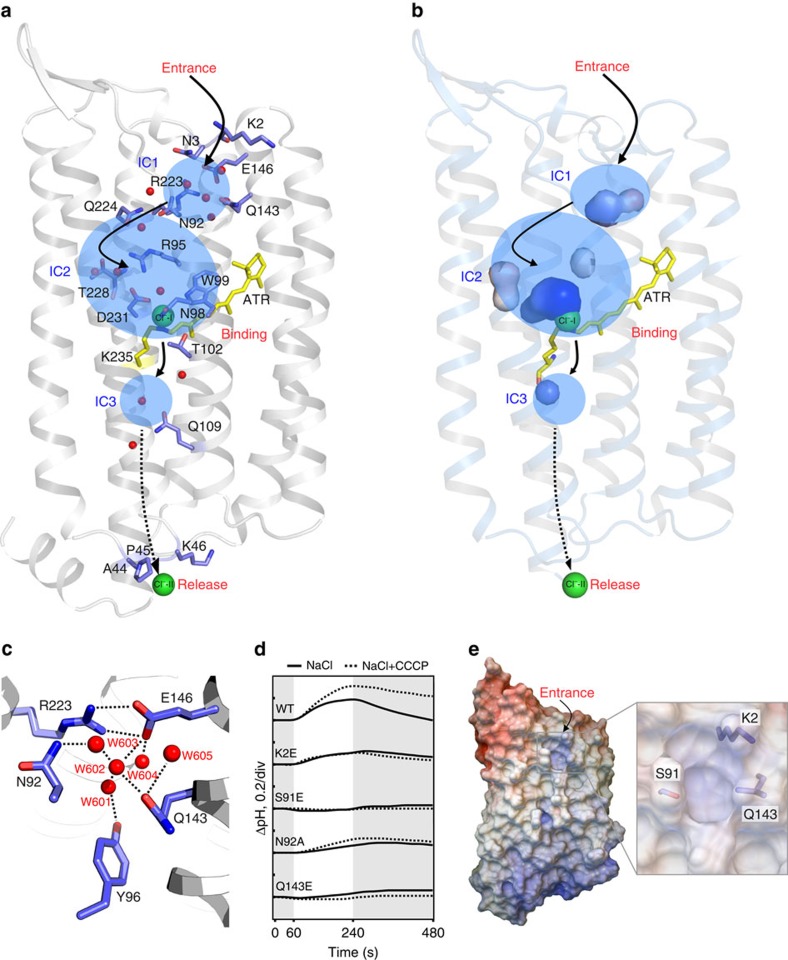
Chloride ion conductance pathway in ClR. (**a**) Three internal cavities (IC1–IC3) inside the ClR structure are highlighted in transparent blue. Water molecules inside the ClR structure are shown in red spheres and amino-acid residues, which interact with the water molecules are indicated. Proposed chloride ion conductance pathway in ClR is shown in arrows (beyond water molecules, dashed arrow). The chloride ions bound to ClR and ATR are shown in green spheres and a yellow stick model. ClR is in the same orientation as Fig. 1a. (**b**) Cavity surface view of ICs in **a**. (**c**) Magnified view of IC1. (**d**) Light-induced pump activities of wild-type (WT) ClR and its mutants at Lys2, Ser91, Asn92 and Gln143. The pumping activities are measured in NaCl (lines) and NaCl with carbonyl cyanide *m*-chlorophenylhydrazone (CCCP; dashed lines). Grey and white sections indicate dark and light conditions, respectively. (**e**) Surface electrostatic potential of ClR. A putative chloride ion entry hole is magnified.

In the crystal structure, ClR adopts a conformation in which the extracellular side is open to the solvent area, whereas the cytosolic side is occluded from the solvent area. This conformation of ClR is similar to that of the HR structures^7^ but quite different from the structure of NaR from *D. eikasta*^13^,^14^. The chloride ion-binding sites of ClR, the locations of the ordered water molecules inside ClR and the electrostatic potential of ClR on its extracellular and cytosolic surfaces allowed us to suggest a potential chloride ion conductive pathway in ClR (Fig. 5). Water molecules inside the ClR structure indicate the presence of three internal cavities (IC1, IC2 and IC3; Fig. 5a,b). IC1, which is below the extracellular surface of ClR, is formed by Asn92, Tyr96, Gln143, Glu146, Arg223 and five well-ordered water molecules, W601–W605 (Fig. 5c). W601–W604 are connected by hydrogen bonds and positioned at the central part of IC1, and all five water molecules interact with the nearby amino-acid residues forming IC1. The amino-acid residues composing IC1 are highly conserved between ClR and NaR^13^,^14^ (Fig. 5c and Supplementary Figs 2 and 6a). Comparison of the two structures shows that IC1 in ClR contains two more water molecules and is larger than that in NaR (Supplementary Fig. 6a). Thus, the ClR structure is open more to the extracellular side than the NaR structure. Rotation of Asn92 appears to be responsible for making IC1 larger in ClR than in NaR and allow a space for the network of water molecules (Supplementary Fig. 6a). Asn92 is also connected to Gln224 and Arg95 through a water molecule-mediated hydrogen bond and bridges IC1 to IC2 of ClR (Fig. 5a). Because the conformational change of Asn92 appears to be important for the formation of IC1 and Asn92 interacts with Arg95, the conserved residue in all microbial rhodopsins and essential for chloride transport in HR^21^, the N92A mutant was prepared to assess the role of Asn92 in chloride ion transport. The pumping activity of ClR expressed in *E. coli* was substantially affected by the N92A mutation (Fig. 5d), supporting the hypothesis that Asn92 is important for chloride transport by ClR during the photocycle, probably in the appropriate formation of IC1.

IC1 of ClR is surrounded by the N-terminal helix and TM C–TM G (Figs 1 and 5a). A hole-like structure is present at the interface of the N-terminal helix and TMs C and D, through which IC1 is connected to the solvent space on the extracellular side (Fig. 5e). This area of ClR, which shows a strong positively charged electrostatic patch, is located outside of the hydrophobic membrane core region (Fig. 5e and Supplementary Fig. 6b)^22^. Therefore, this could be the point that the chloride ion enters the inside of ClR, and the positive-electrostatic potential may help lead chloride ions into IC1. To test this hypothesis, we prepared mutants K2E, S91E and Q143E to introduce a bulky, negatively charged side chain into the amino acids around the hole. The pumping activity of ClR expressed in *E. coli* was almost shut down in the mutants S91E and Q143E and significantly affected by the K2E mutation (Fig. 5d), suggesting that the bulky negatively charged side chain affects the activity of ClR by physically and electrostatically inhibiting the entry of chloride ions into the hole.

Four water molecules (W502–W505) and one chloride ion (Cl^−^-I) are observed in IC2 (Fig. 5a and Supplementary Fig. 6c). The water molecules form a hydrogen bond network with Thr228, Arg95 and Asp231. Chloride ions are likely to reach the Cl^−^-I-binding site through IC2. IC3 is formed around Gln109 of the ‘NTQ motif' on the cytosolic side of the retinal (Fig. 5a,b and Supplementary Fig. 6d). Two water molecules in IC3 interact with neighbouring amino-acid residues Ala50, Ser54, Gln109, Lys235 and Thr242. Another water molecule, which interacts with Trp201 and Ser234, is observed between the retinal and IC3. Beyond IC3 in ClR, there are no more water molecules on the cytosolic side indicating that the cytosolic side of ClR is closed.

In the NaR structure, another internal cavity composed of the amino acid residues on TM A and TM B (Tyr45, Thr49, Asn52, Ser60, Asn61, and Ser64) was observed just below the cytosolic surface^13^,^14^. It is unclear whether ClR also forms an internal cavity corresponding to this cavity in NaR and the conducting chloride ion passes through this cavity during the photocycle because the residues constituting the cavity are not conserved between ClR and NaR (Supplementary Fig. 2).

## Discussion

The ClR crystal structure presented in this work together with the recently reported NaR structures^13^,^14^ reveals the molecular architectures of novel light-driven ion pumps in marine bacteria. ClR contains an NTQ motif in the putative ion conductive pathway and functions as an inward light-driven chloride pump, whereas NaR's motif is NDQ (aspartate replaces threonine) and functions as an outward sodium pump. Notably, in halophilic archaeal rhodopsin pumps, a single-site mutation of Asp85 of BR, which is the counterion residue of PSB, to Thr changes BR, an outward proton pump, to an inward chloride pump like HR^23^. HR can also be converted into a proton pump in the presence of anionic azide^24^,^25^. The HR and BR crystal structures showed that the chloride ion-binding site at PSB of HR corresponds to the position of the negatively charged side chain of Asp85 of BR^7^,^26^, suggesting that BR and HR use a common mechanism for ion conductance and that the chloride ion at PSB of HR compensates for the negative charge from Asp85 of BR^27^,^28^. The ClR crystal structure shows a chloride ion-binding site (Cl^−^-I) at PSB of the NTQ rhodopsin, which suggests that balancing the negative charge at PSB might also be an important principle for chloride ion transport in NTQ rhodopsins. Superimposition of the ClR and NaR structures shows that Cl^−^-I in ClR corresponds to the location of the side chain of Asn112 in NaR, but not to the side chain of Asp116, the counterion of PSB. From our results on ClR, the structural and functional analyses of NaR^13^,^14^, and the studies on HR^7^ and BR^3^, we hypothesize the chloride pumping mechanism by ClR (Supplementary Fig. 7). (1) In the resting state, ClR is open to the extracellular side and IC1, IC2 and IC3 are formed as in the ClR crystal structure. A chloride ion enters inside ClR through IC1 and reaches the binding site at PSB (the binding site of Cl^−^-I) in IC2; (2) absorption of light induces isomerization of all-*trans* retinal to 13-*cis* configuration; (3) the chloride ion moves to the cytosolic side; (4) structural changes of ClR cause ClR open to the cytosolic side to form IC4. Whether a movement of helix F of ClR is involved in the formation of a cytosolic IC as suggested in NpHR^29^ is an open question; (5) the chloride ion moves through IC4 to the binding site on the A–B loop (the binding site of Cl^−^-II) and releases to the cytosolic side; (6) ICs are rearranged to the resting state. Investigations into whether ClR and NaR are inter-convertible, as with HR and BR, and whether the chloride and sodium-binding sites are related in ClR and NaR may increase our understanding regarding the molecular mechanisms of these new rhodopsin pumps, as well as the evolutionary relationship between them.

Phylogenetic analysis suggests that ClR and HR have independently evolved into light-driven chloride pumps in marine bacteria and halophilic archaea, respectively^10^. The differences in the sequences of key amino-acid residues of these pumps, which are potentially involved in chloride ion conductance and represented by the NTQ motif of ClR and the TSA motif of HR, raises an intriguing question how ClR and HR convergently evolved at the molecular level. The ClR crystal structures presented in this report show that most of the amino-acid residues constituting the internal cavities of ClR are different from those of HR (Supplementary Fig. 6). Lys235, which is attached to the retinal by a Schiff base linkage, as well as Arg95 and Asp231, a counterion pair essential for the activity of ion-pumping rhodopsins, are the few amino-acid residues of the internal cavities conserved between ClR and HR. Despite the differences in their amino-acid sequences and structures at their active centres, the binding of a chloride ion at PSB of ClR (Cl^−^-I; Figs 1 and 3) strongly suggests that ClR might use a chloride transport mechanism similar to that used by HR. The chloride-binding site at the A–B loop of ClR (Cl^−^-II) was unexpected because the ClR structure appeared to adopt a closed conformation on the cytoplasmic side. We speculate that the binding and release of a chloride ion at Cl^−^-II might assist the vectorial transport of chloride ions into cells from the extracellular side. A spectroscopic study of FR, another NTQ rhodopsin having 39% amino-acid sequence identity with ClR, suggested that FR has another chloride ion-binding site at the O-intermediate on the extracellular side and that a chloride ion is accepted by FR on the formation of the O-intermediate, whereas the O-intermediate of HR disappears on acceptance of a chloride ion^16^. The Cl^−^-II-binding site of ClR is unlikely to be the chloride-binding site suggested by the spectroscopic study of FR. Parallel and comparative studies of ClR and HR would lead to an understanding of the chloride ion transfer mechanisms used by light-driven ion-pumping rhodopsins. This understanding is essential for the rational design of optimal light-driven ion pumps for optogenetic applications.

## Methods

### Cloning, protein expression and purification

The ClR gene (GI: 594833795) was amplified by PCR using primers (5′-CGGAATTCAATGAAAAATATTGAAAGCTTATTT-3′ and 5′-CCGCTCGAGGGCTGCTTTAGAGTCCATACCTATACGCCCT-3′) from genomic DNA extracted from *N. marinus* S1-08^T^ cells and cloned into a pET21b vector to append a (His)_6_-tag at its C terminus. The plasmid was used to transform *E. coli* BL21-CodonPlus (DE3; Agilent Technologies), and the transformed cells were grown in YT media at 37 °C. When the OD_600nm_ was ∼0.6, the medium was supplemented with 50 μM all-*trans* retinal (Sigma Aldrich) and 0.5 mM isopropyl-β-D-thiogalactoside (IPTG) was added to induce ClR expression for 6 h at 30 °C. Cells were lysed by sonication in buffer containing 50 mM Tris-HCl (pH 7.0) and 150 mM NaCl. The membrane fraction was isolated by ultracentrifugation at 142,000*g* for 40 min at 4 °C. The pellet containing membrane proteins was resuspended in buffer containing 50 mM Tris-HCl (pH 7.0), 150 mM NaCl, 20 mM imidazole, 1% *n*-dodecyl-β-D-maltoside (DDM) and 0.2% cholesteryl hemisuccinate and kept for 2 h at 4 °C to solubilize it. The resuspended protein was purified by TALON affinity chromatography, and then the eluate was applied to a Superdex-200 size-exclusion column (GE Healthcare) equilibrated with buffer containing 20 mM HEPES (pH 7.5), 150 mM NaCl, 0.05% DDM and 0.01% cholesteryl hemisuccinate for further purification. ClR mutant proteins, T102N and T102D, were purified using the same procedure for wild-type ClR.

### Crystallization and data collection

The ClR protein for crystallization trials was concentrated to 50 mg ml^−1^ and applied for an *in meso* approach as follow. The protein solution was mixed with monoolein (1-oleoyl-rac-glycerol) in a 1:1.5 protein to lipid ratio (w/w) using a syringe lipid mixer (Hamilton) at 20 °C for the lipid cubic phase formation. After a clear lipidic cubic phase formed, the mix was dispensed onto sandwich plates in 50 nl drops overlaid with 700 nl precipitant solution using a LCP Mosquito robot (TTP Labtech). Type A crystals grew within 2 weeks at 17 °C in 30% PEG, 500 DME, 0.1 M sodium chloride, 0.1 M MES (pH 6.0) and 0.1 M calcium chloride dehydrate. Type B crystals grew in 30% PEG 400, 0.1 M sodium chloride, 0.1 M sodium acetate (pH 4.5) and 0.1 M magnesium chloride. T102N and T102D, two ClR mutants, were crystallized in the same condition as the type B crystal. To create an anomalous difference Fourier map of Br^−^, the crystallization mixtures used to obtain the native crystals were supplemented with 200 mM NaBr. Diffraction data for type A and two mutants of ClR crystals were collected on the 5C beamline at Pohang Accelerator Laboratory (Pohang, South Korea) using a 75 μm diameter micro-beam. Diffraction data for type B crystals were collected on the BL1A beamline at the Photon Factory (Tsukuba, Japan), using a 0.03 × 0.01 mm micro-beam. The diffraction data were processed with XDS software^30^. Type A crystals belonged to space group C121 and contained one molecule per asymmetric unit. Type B crystals belonged to space group I121 and contained one molecule per asymmetric unit.

### Structure determination and refinement

The structure was solved by molecular replacement using the KR2 structure (PDB ID: 3X3B)^13^ as a search model using Molrep^31^ in the CCP4 suite^32^. The resultant structure was refined by iterative rounds of rigid body, *xyz* coordinates and individual b-factor with Refmac^33^ and Phenix^34^, and manually adjusted with Coot^35^ resulting in final *R*_work_/*R*_free_ of 15.40/19.38% for type A crystal and 17.84/22.24% for type B crystal. For anomalous difference Fourier maps, the diffraction data sets collected at the peak absorption wavelength of Br^−^, 0.9197 Å, were processed to retain anomalous signals and the map was generated using Phenix^33^.

### CPM stability assay

The CPM stability assay was performed as follow^36^. A 4 mg ml^−1^ stock solution of CPM dye (Invitrogen) was prepared in dimethylsulfoxide (Sigma) and diluted to 0.1 mg ml^−1^ in dilution buffer (20 mM HEPES (pH 7.5) and 150 mM NaCl) before use. The thermal denaturation assay was performed in a total volume of 130 μl. The ClR protein and its mutants (F15A, W72A, Y83A and delta C-terminal helix; 1–254) were diluted in the appropriate buffer to a final volume of 120 μl. Ten microlitre of the diluted dye was added and thoroughly mixed with the protein. The reaction mixture was transferred within a 5 min period to a sub-micro quartz fluorometer cuvette (Stana Cells, Inc., Atascadere, CA) and heated in a controlled way with a ramp rate of 2 °C min^−1^ in a Cary Eclipse Spectrofluorometer. The excitation wavelength was set at 387 nm, and the emission wavelength was set at 463 nm. Assays were performed over a temperature range beginning at 30 °C and ending at 90 °C. The stability data were processed using GraphPad Prism software (GraphPad Software, San Diego, CA).

### Measurement of the pumping activities of ClR and its variants

*E. coli* BL21-CodonPlus cells expressing ClR or its variants were incubated at 37 °C in YT media supplemented with 100 mg ml^−1^ ampicillin and 10 mg ml^−1^ chloramphenicol. When the OD_600nm_ reached ∼0.8, ClR or its mutant expression was induced by adding 0.5 mM IPTG and 50 μM all-*trans*-retinal. After additional cultivation for 6 h at 30 °C, cells were collected by centrifugation (4,000*g* for 5 min), washed three times with 100 mM NaCl or 100 mM Na_2_SO_4_, and then suspended in desired solvents for measurement by adjusting the OD_600nm_ value to ∼8.2. Cell suspensions were placed in the dark for over an hour, and then proton ion flux changes in the buffer were measured using a pH electrode (Mettler Toledo, Switzerland). The light source used was a 100 W halogen lamp (Osram, Germany).

### Ultraviolet–visible spectroscopy

The purified wild-type ClR and two mutant, T102N and T102D, samples were divided into two parts and buffer-exchange was accomplished to 1% DDM in deionized water for one half and 1% DDM in 1.5 M NaCl for the other using PD midiTrapTM G-25 column (GE Healthcare). The absorption spectra were scanned for the protein samples using a V-650 spectrophotometer (JASCO).

### Data availability

Coordinates and structure factors have been deposited in the Protein Data Bank under accession codes 5G28 (pH 6.0 crystal, type A), 5G54 (pH 4.5 crystal, type B), 5G2A (bromide soaked crystal), 5G2D (T102N crystal) and 5G2C (T102D crystal). Remaining data are available from the corresponding authors on reasonable request.

## Additional information

**How to cite this article:** Kim, K. *et al.* Crystal structure and functional characterization of a light-driven chloride pump having an NTQ motif. *Nat. Commun.* 7:12677 doi: 10.1038/ncomms12677 (2016).

## Supplementary Material

Supplementary Information Supplementary Figures 1-7 and Supplementary References.

## Figures and Tables

**Table 1:** Data collection and refinement statistics.

PDB code	5G28* (type A crystal)	5G54* (type B crystal)	5G2A* (Br ion crystal)	5G2D* (T102N)	5G2C* (T102D)
*Data collection*					
Space group	C121	C121	C121	C121	C121
*a*, *b*, *c* (Å)	102.76, 49.40, 69.33	102.72, 49.43, 77.03	102.77, 48.85, 69.36	103.25, 49.45, 78.40	103.44, 50.03, 77.79
*α*, *β*, *γ* (°)	90, 109.85, 90	90, 131.16, 90	90, 110.13, 90	90, 131.89, 90	90, 131.47, 90
Resolution (Å)	65.21–1.57 (1.62–1.57)†	40.00–2.0 (2.05–2.0)†	65.13–2.17 (2.21–2.17)†	58.36–1.80 (1.83–1.80)†	58.28–2.31 (2.34–2.31)†
*R*_merge_ (%)	11.1 (65.9)†	10.2 (69.3)†	5.9 (20.9)†	7.0 (39.3)†	6.0 (31.3)†
*I/σI*	30.81 (4.23)†	11.1 (1.65)†	35.46 (7.59)†	22.67 (2.57)†	14.43 (1.86)†
Completeness (%)	97.4 (93.06)†	99.3 (96.4)†	99.0 (98.8)†	97.1 (81.9)†	94.5 (73.5)†
Redundancy	4.4 (2.7)†	5.6 (4.9)†	5.8 (3.9)†	4.6 (2.4)†	4.3 (2.0)†
					
*Refinement*					
Resolution (Å)	65.21–1.57	40.0–2.0	65.13–2.17	58.36–1.80	58.28–2.31
No. of reflections	42,520	18,695	16,316	25,345	11,889
*R*_work_/*R*_free_ (%)	15.40/19.38	17.60/22.52	15.31/20.71	17.51/20.52	19.45/23.88
					
*No. of atoms*					
Protein	2,072	2,105	2,072	2,095	2,101
Retinal	20	20	20	20	20
Water	136	84	59	93	15
Lipid fragments	99	101	—	108	108
Chloride	2	2	2	2	1
					
B *factors (Å*^*2*^)					
Protein	23.82	19.03	29.22	22.87	29.27
Retinal	15.57	11.06	21.66	17.02	22.14
Water	39.86	34.65	40.69	40.63	37.52
Lipid fragments	48.21	44.54	—	48.38	47.48
Chloride ions	32.77	38.75	Br^−^ (67.85)	40.97	58.43
					
*r.m.s. deviations*					
Bond lengths (Å)	0.026	0.018	0.021	0.023	0.019
Bond angles (°)	1.92	1.98	1.83	1.90	1.91
Crystal size (μm)	50 × 50 × 50	30 × 30 × 30	50 × 50 × 50	50 × 50 × 50	50 × 50 × 50
Radiation dose (MGy)	0.04	0.12	0.04	0.04	0.04

PDB, Protein Data Bank; r.m.s., root mean squared.

^*^Number of crystal used for data collection: 1.

^†^Highest-resolution shell is shown in parenthesis.
