# Supplementary material for: Crystal structure and functional characterization of a light-driven chloride pump having an NTQ motif
Source: Nat Commun. 2016 Aug 24;7:12677. doi: 10.1038/ncomms12677 (PMC4999514; doi:10.1038/ncomms12677)
Supplement: Supplementary Information — Supplementary Figures 1-7 and Supplementary References. [file ncomms12677-s1.pdf]

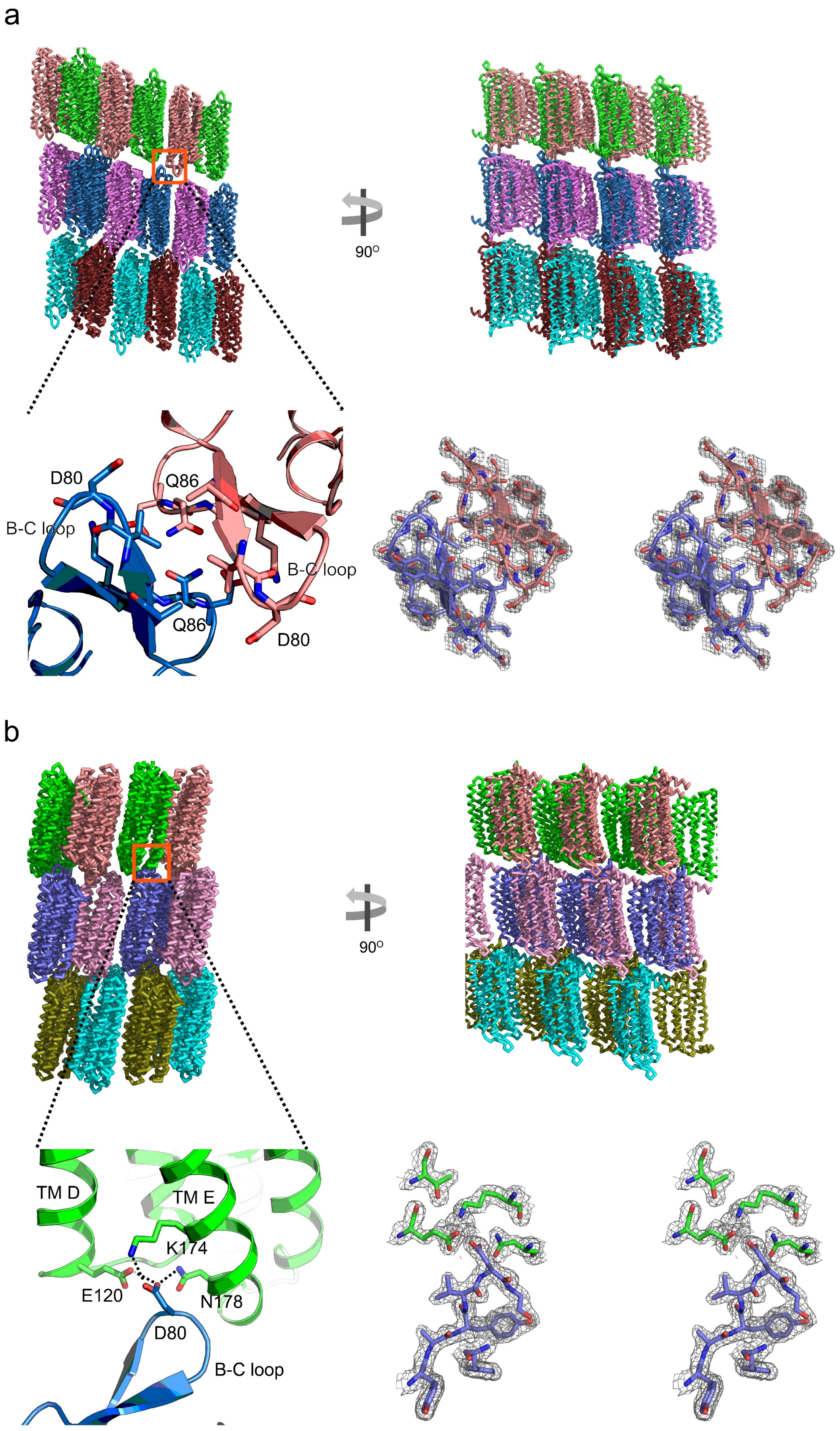

Supplementary Figure 1 Crystal packing of ClR and electron density maps. Crystal packing of type A crystal (**a**) and type B crystal (**b**). Crystal contacts at B-C loop are magnified and stereo view of σ^A-weighted 2Fo-Fc maps (gray meshes contoured at 1.5σ) are shown.

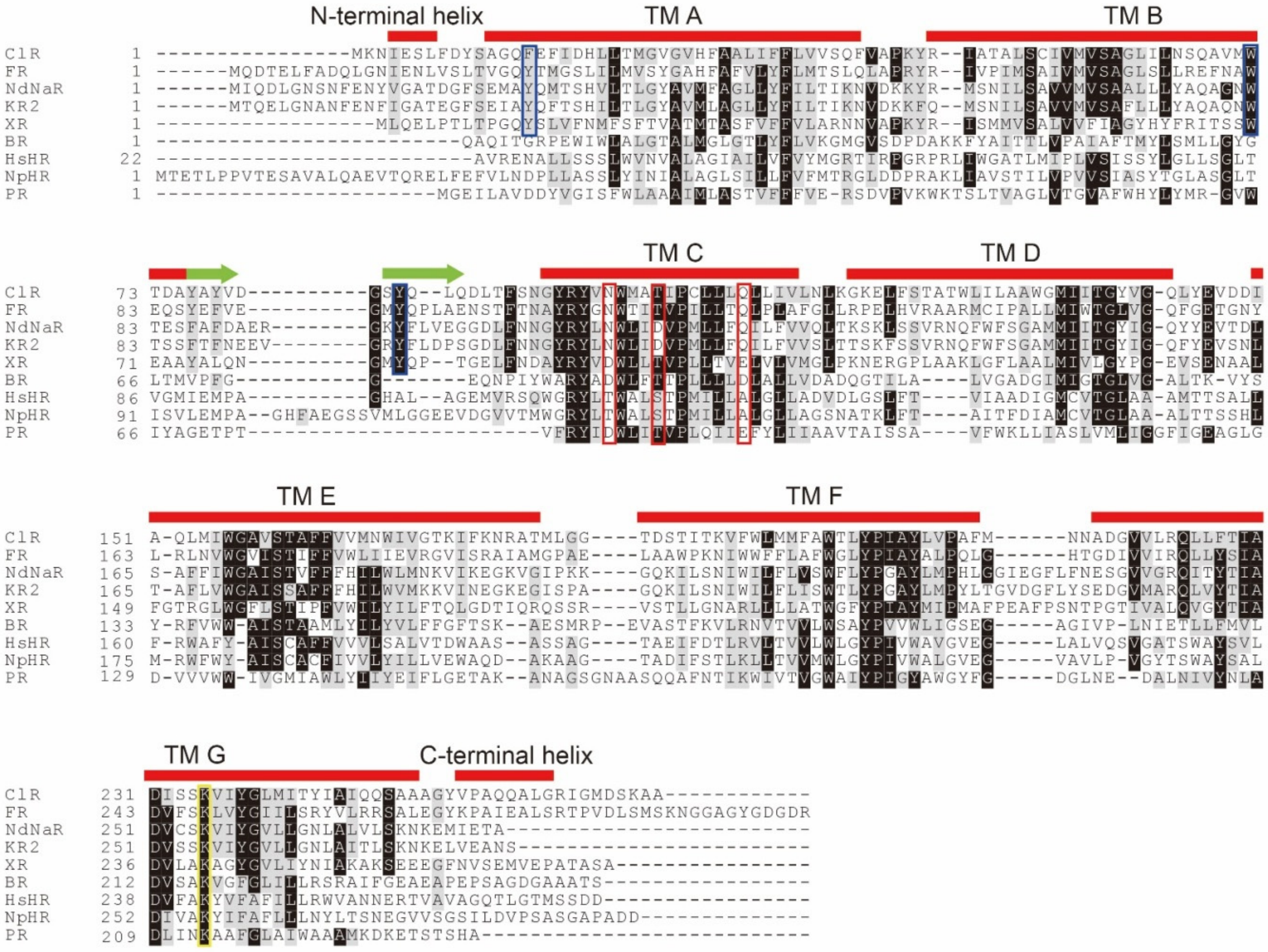

Supplementary Figure 2 Structure-guided sequence alignments of light-driven ion-pumping microbial rhodopsins. Secondary structure elements are based on the ClR structure and represented by red-rectangles for α-helices and green arrows for β-strands. Identical and similar residues are highlighted in black and gray, respectively. The conserved amino acid residues forming the 3 omega motif (blue-open rectangles), the NTQ motif (or corresponding residues in other rhodopsins) (red-open rectangles), and Schiff base linkage (yellow-open rectangle) are indicated. Chloride-pumping rhodopsins from *Nonlabens marinus* (ClR) and *Fulvimarina pelagi* (FR), sodium-pumping rhodopsins from *Nonlabens dokdonensis* (NdNaR) and *Dokdonia eikasta* (*Krokinobacter eikastus*) (KR2), xanthorhodopsin (XR), bacteriorhodopsin (BR), halorhodopsins from *Halobacterium salinarum* (HsHR) and *Natronomonas pharaonis* (NpHR), and proteorhodopsin (PR).

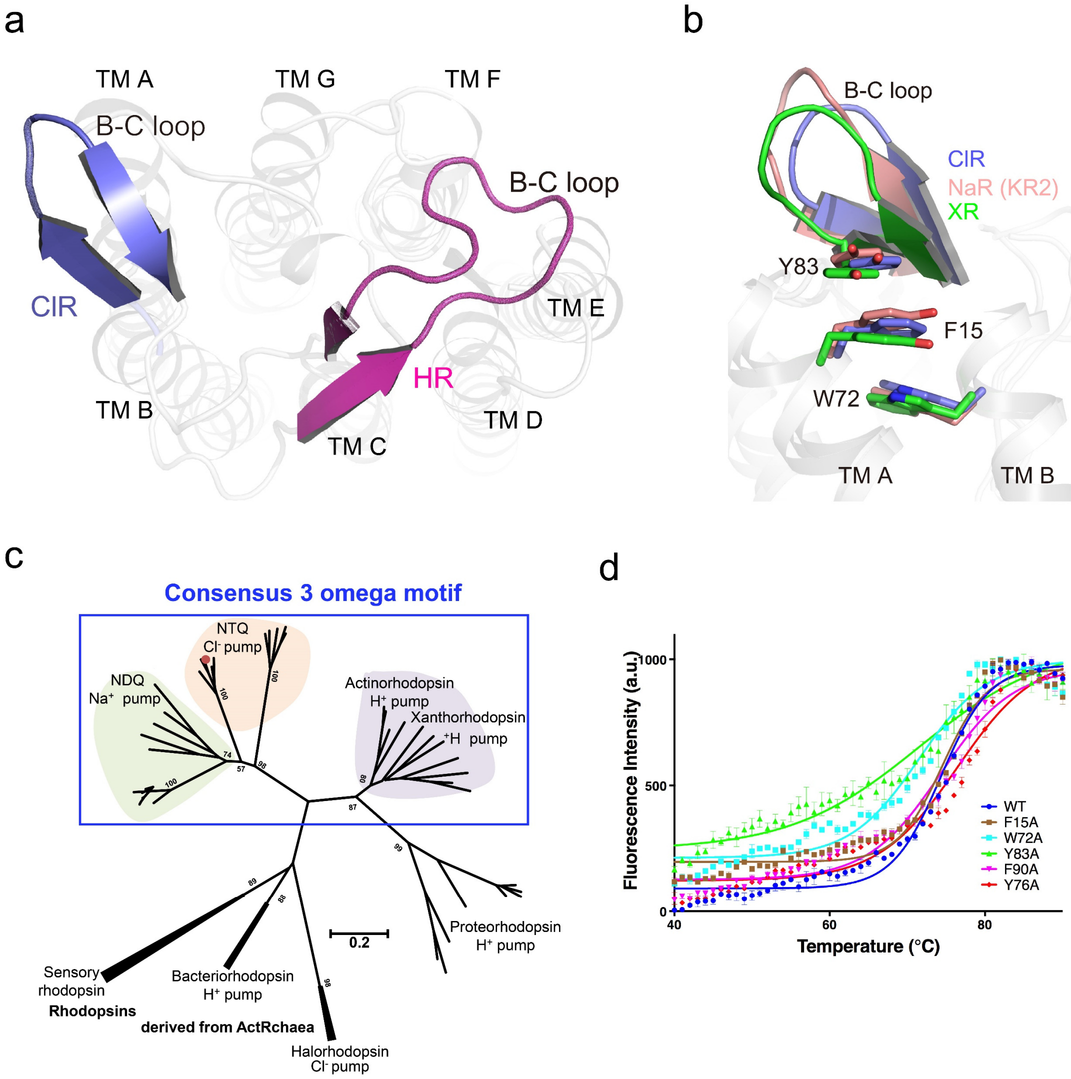

Supplementary Figure 3 3 omega motif and orientation of B-C loop. (**a**) Comparison of B-C loop conformation between ClR and HR. Crystal structures of ClR and HR (PDB ID: 1E12)[1] are superimposed and the B-C loops of ClR and HR are depicted in blue and pink, respectively. (**b**) 3 omega motives and B-C loops of ClR (blue), NaR (KR2) (salmon, PDB ID: 3X3B)[2], and XR (green, PDB ID: 3DDL)[3]. (**c**) Phylogenetic relationship among microbial rhodopsins. Microbial rhodopsins containing the conserved 3 omega motif are enclosed by a blue-rectangle. (**d**) Fluorescence thermal stability profiles of wild-type (WT) and mutant ClRs (mutations in 3 omega motif). Tm values are WT, 74°C; F15A, 74°C; W72A, 71°C; and Y83A, 72°C. F90A and Y76A are negative control and two mutants are no difference compared to the wild type. The curves are representative of three experiments and the error bars represent the mean ± s.d. a.u., arbitrary units.

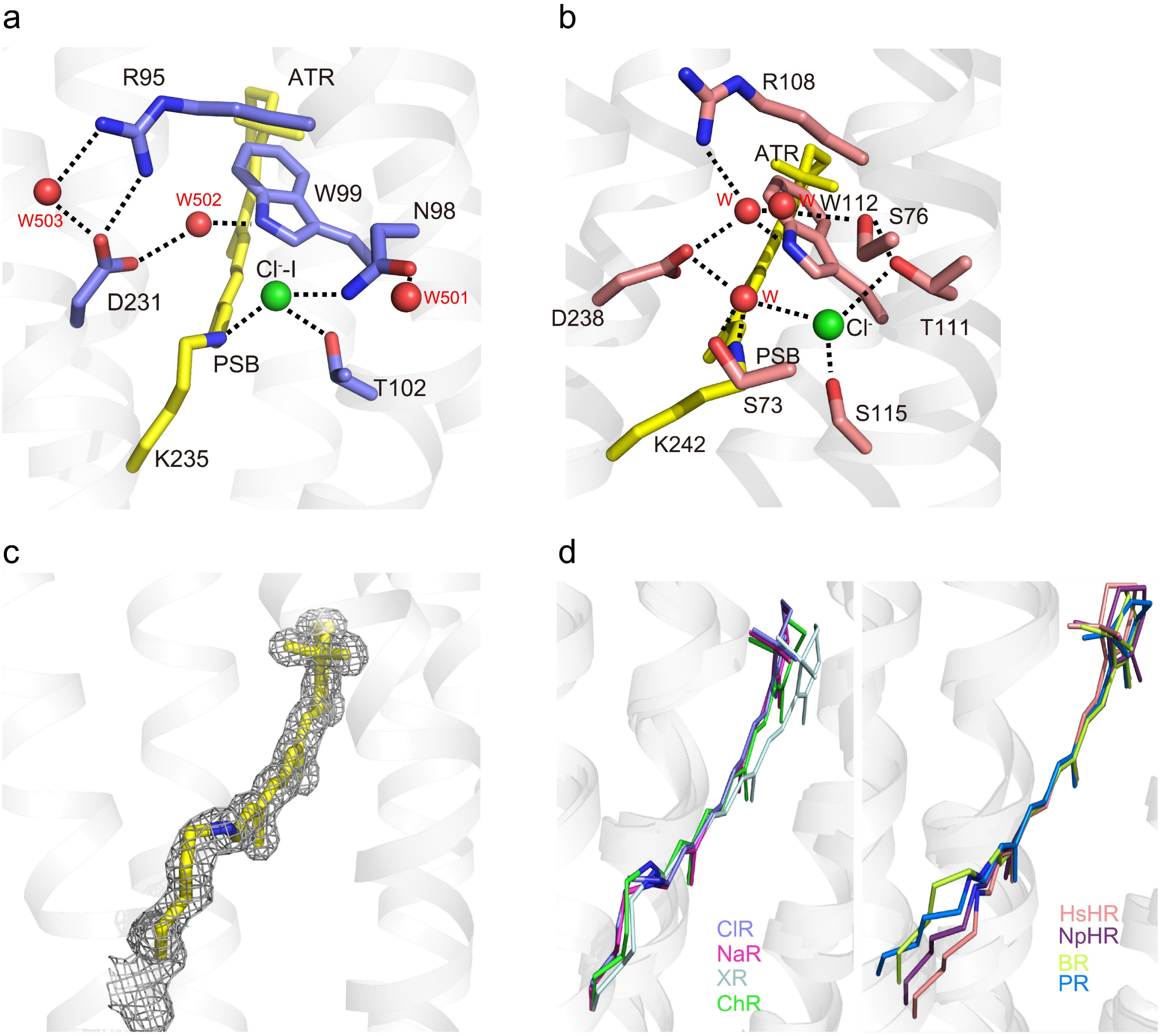

Supplementary Figure 4 Comparison of active center structures between ClR (**a**) and HR (**b**). Chloride ion and water molecules are shown by green and red spheres, respectively. Hydrogen-bonds are shown in dashed lines and ATR is shown in yellow stick model. **(c)** K235-all *trans*-retinal (yellow stick model) shown in 2Fo-Fc map (contoured at 1.5σ). **(d)** Superimposition of lysine-ATR of ClR (sky blue), NaR (3X3B, magenta), XR (3DDL, grey), ChR (3UG9, green), HsHR (1E12, salmon), NpHR (3A7K, purple), BR (1QKO, lemon) and PR (4JQ6, marine blue).

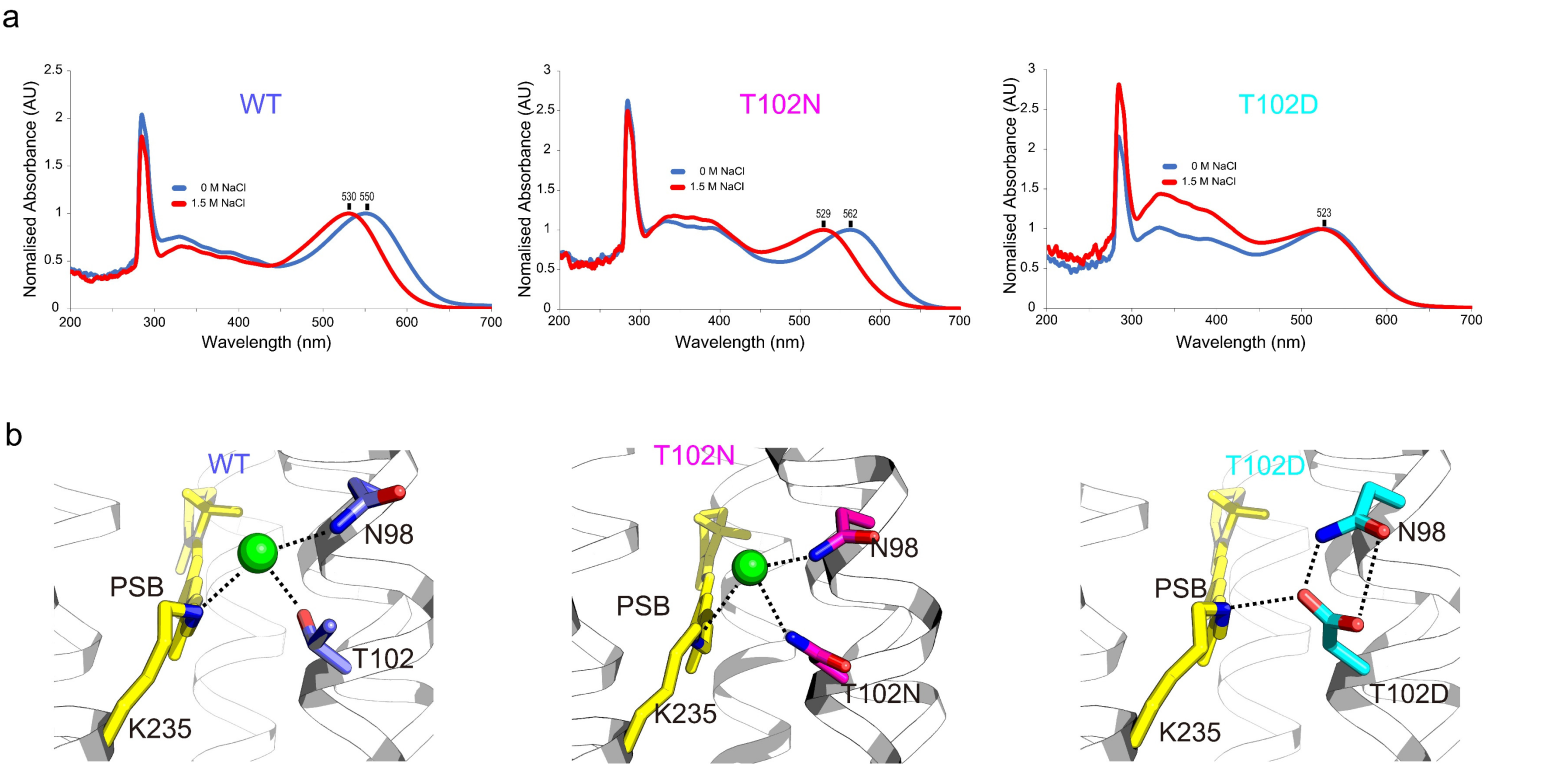

Supplementary Figure 5 Absorption spectra and structures at PSB. (**a**) Absorption spectra of wild-type (WT) and mutants of ClR (T102N and T102D) in a solvent without (blue) and with NaCl (red). (**b**) Comparison of structures at PSB among wild-type ClR and mutants.

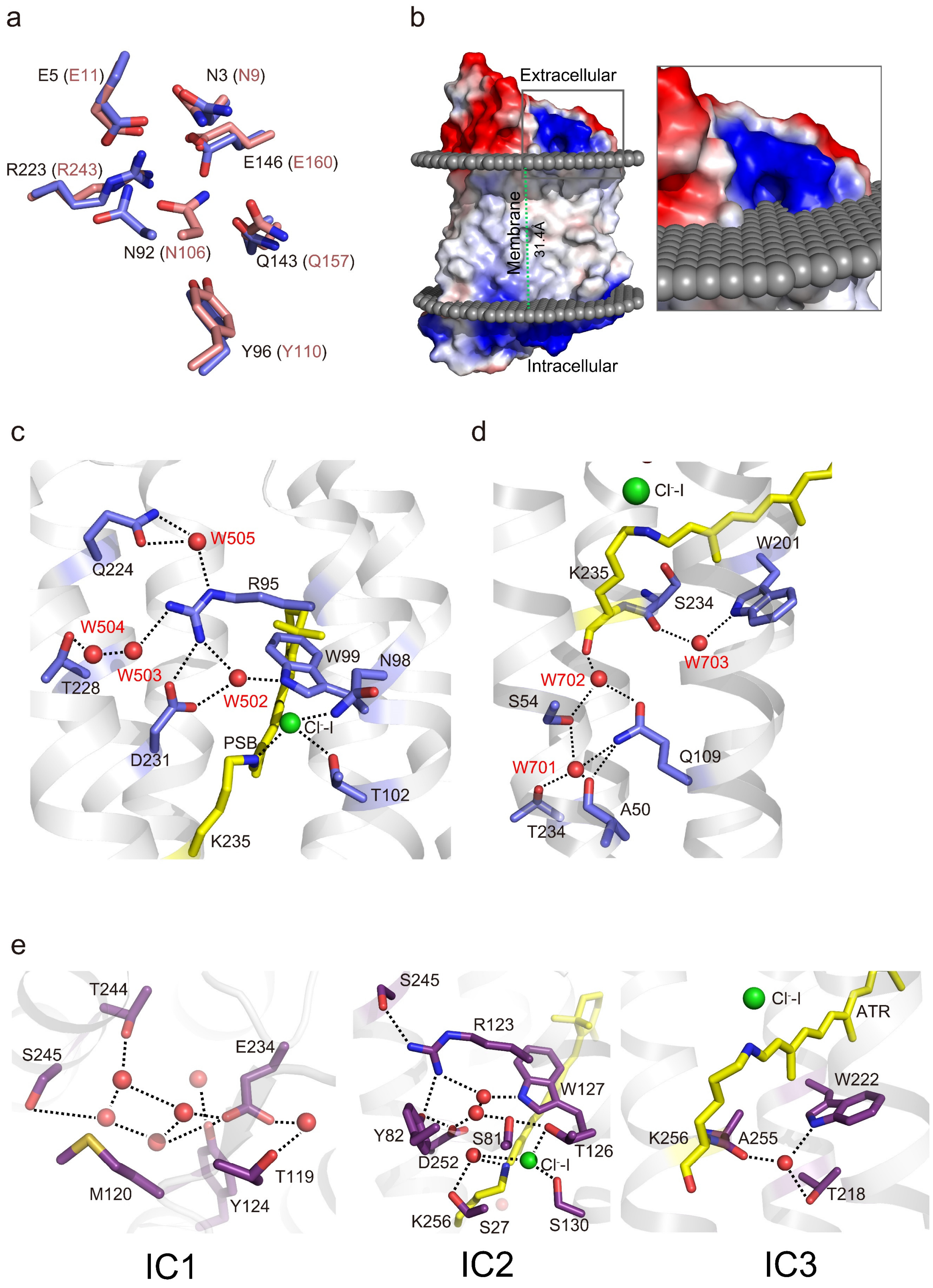

Supplementary Figure 6 Internal cavities (ICs) of ClR structure. (**a**) Comparison of IC1 of ClR (blue) and NaR (KR2) (pink, PDB ID: 3X3B)[2]. (**b**) A putative chloride ion entry hole connected to IC1 of ClR and the hydrophobic membrane core boundaries calculated with the PPM server[4]. (**c**) and (**d**), IC2 and IC3 of ClR, respectively. (**e**) Internal cavities (ICs) of NpHR (3A7K). IC figures are in the same orientation as those of ClR. Red spheres and dashed lines represent water molecules and hydrogen-bonds.

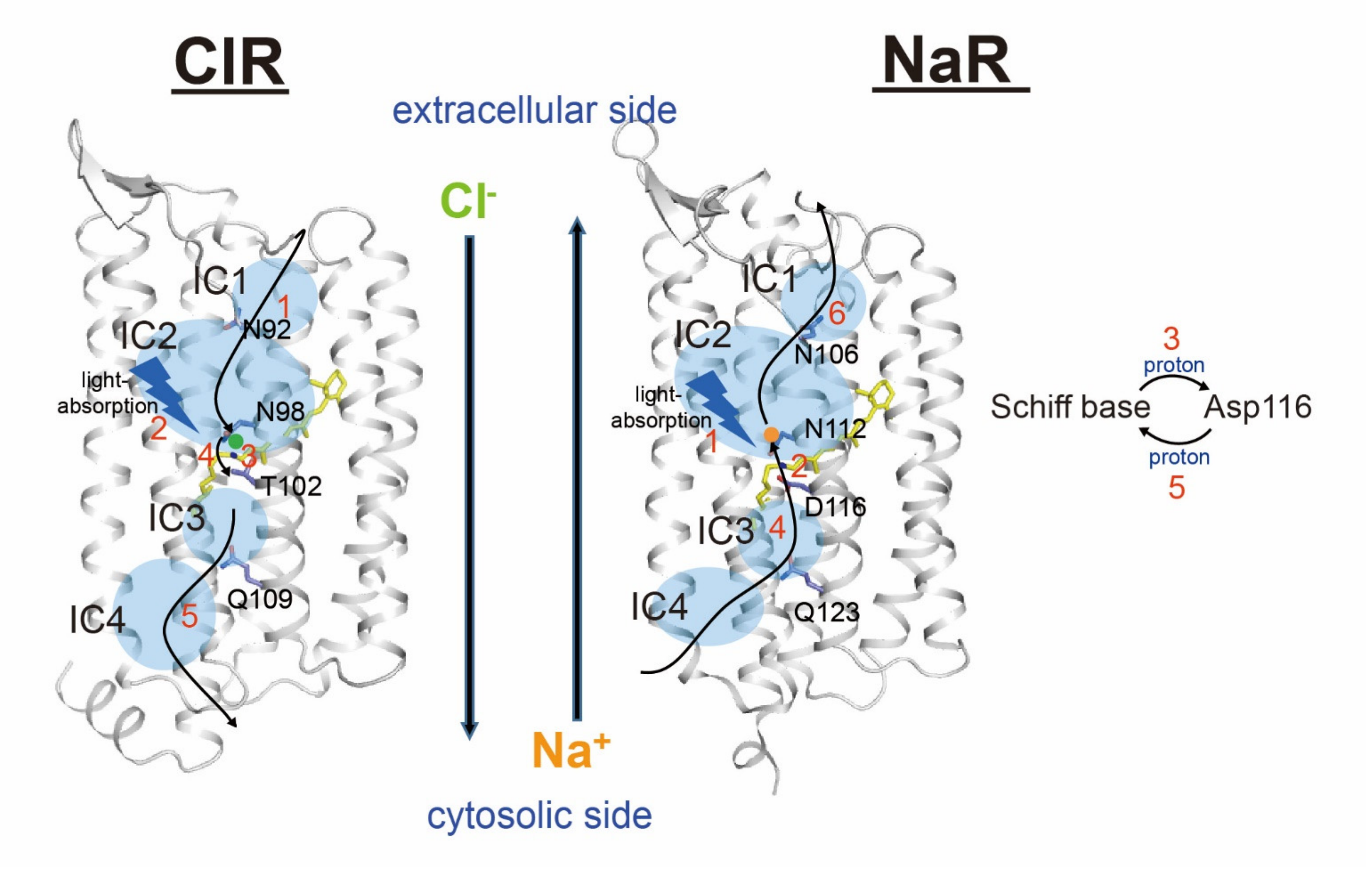

Supplementary Figure 7 Models of ion translocation by ClR and NaR. The sequence of light-absorption, isomerization of retinal, and ion transfer steps are indicated by numbers in red. The sodium transfer model by NaR was proposed in the previous studies[2,5]. Key amino acid residues and retinal are shown in sticks model. Four internal cavities (IC1-IC4) inside the ClR and NaR structures are highlighted in transparent blue. Chloride ion and sodium ion are shown by green and orange spheres, respectively.

Supplementary References

1. Kolbe, M., Besir, H., Essen, L.O. & Oesterhelt, D. Structure of the light-driven chloride pump halorhodopsin at 1.8 Å resolution. *Science* **288**, 1390–1396 (2000).
2. Kato, H.K. *et al.* Structural basis for Na^+ transport mechanism by a light-driven Na^+ pump. *Nature.* **521**, 48–55 (2015).
3. Luecke, H. *et al.* Crystallographic structure of xanthorhodopsin, the light-driven proton pump with a dual chromophore. *Proc. Natl. Acad. Sci. USA.* **105**, 16561–16565 (2008).
4. Lomize, M.A., Pogozheva, I.D., Joo, H., Mosberg, H.I. & Lomize, A.L. OPM database and PPM web server: resources for positioning of proteins in membranes. *Nucleic. Acids. Res.* **40**, D370–D376 (2012).
5. Gushchin, I. *et al.* Crystal structure of a light-driven sodium pump. *Nat. Struct. Mol. Biol.* **22**, 390–396 (2015).
